# Patient and public involvement in cancer research: A scoping review

**DOI:** 10.1002/cam4.6200

**Published:** 2023-06-16

**Authors:** Sara Colomer‐Lahiguera, Matthieu Steimer, Ursula Ellis, Manuela Eicher, Margaret Tompson, Tourane Corbière, Kristen R. Haase

**Affiliations:** ^1^ Institute of Higher Education and Research in Healthcare (IUFRS), Faculty of Biology and Medicine, University of Lausanne (UNIL), Lausanne University Hospital (CHUV) Lausanne Switzerland; ^2^ Master of Advanced Studies in Public Health student Institute of Global Health, Geneva University Geneva Switzerland; ^3^ Woodward Library University of British Columbia Vancouver Canada; ^4^ Saskatchewan Canada; ^5^ School of Nursing University of British Columbia Vancouver Canada

**Keywords:** cancer, patient and public involvement, PPI, research

## Abstract

**Introduction:**

Patient and public involvement (PPI) in research emphasizes the importance of doing research with, rather than for people with lived health/illness experience(s). The purpose of this scoping review is to investigate the breadth and depth of scientific literature on PPI in cancer research and to identify how is PPI applied and reported in cancer research.

**Methods:**

We searched MEDLINE, Embase, CINAHL, and PsycInfo up to March 2022. All titles/abstracts and full‐text results were screened by two reviewers. Data were analyzed and are presented in both narrative and tabular format.

**Results:**

We screened 22,009 titles/abstract, reviewed 375 full‐text articles, of which 101 studies were included in this review. 66 papers applied PPI; 35 used co‐design methodologies. PPI in cancer research in published research has increased steadily since 2015 and often includes those with a past diagnosis of cancer or relatives/informal caregivers. The most common applied methods were workshops or interviews. PPI was generally used at the level of consultation/advisor and occurred mainly in early stages of research. Costs related to PPI were mentioned in 25 papers and four papers described training provided for PPI.

**Conclusions:**

Results of our review demonstrate the nature and extent of PPI expansion in cancer research. Researchers and research organizations entering the fray of PPI should consider planning and reporting elements such as the stage, level, and role type of PPI, as well as methods and strategies put in place to assure diversity. Furthermore, a thorough evaluation of whether all these elements meet the stated PPI purpose will help to grasp its impact on research outcomes.

**Patient or Public Contribution:**

Two patients participated in the stakeholder consultation as part of the scoping review methodology, contributed to the discussion on refining the results, and critically reviewed the manuscript. Both are co‐authors of this manuscript.

## 1 INTRODUCTION

There is growing recognition of the value of patient and public involvement (PPI) in research, and specifically in health research, as a means of improving validity and relevance of research findings.^1^, ^2^, ^3^ While many definitions of PPI exist, in this paper we draw on the definition from the National Institute for Health and Care Research (NIHR), which describes PPI as: “research conducted ‘with’ or ‘by’ members of the public rather than ‘for’ or ‘about’ them”.^4^ PPI approaches recognize the experiential knowledge of people with lived health or illness experiences and posit that the incorporation of such voices in research will improve the effectiveness and value of health research.^5^ Those with lived experience may also bring unique insights to health research by increasing the quality, transparency and relevance of research to patients, improving recruitment and retention rates of research participants, broadening the range of people represented in studies, and improving the dissemination of results beyond the academic setting.^6^, ^7^, ^8^

Numerous models and theoretical frameworks exist to support PPI in research.^9^, ^10^ The incorporation of PPI in health research is particularly well established and supported in the United States, Canada, and the UK by organizations including the Patient‐Centered Outcomes Research Institute (PCORI),^11^ the Strategy for Patient Oriented Research (SPOR),^12^ or the National Institute for Health and Care Research (NIHR),^5^ respectively. PPI conceptualization differs depending on the framework, but in general it is acknowledged as a continuum ranging from lower to higher levels of involvement including information, participation, consultation, collaboration, to (the highest level) (co‐)leading.^5^, ^11^, ^13^, ^14^, ^15^, ^16^, ^17^, ^18^ Overall, PPI in research is still a developing phenomenon in many countries, which can take on different forms, corresponds to diverse practices, and entails multiple rationales.^9^

Different aspects of interest have been highlighted in the literature that need to be considered when implementing a PPI approach in research. First, tokenism (or “tick‐box”) approaches to participation are a major challenge. That is, the inclusion or naming of a patient without their *authentic engagement*. We use italics here to emphasis the problematic nature of the idea of authentic engagement, and how this can be determined given variable preferences of patient partners.^9^ Second, representation of diverse patient profiles in terms of socio‐demographics, economics, education, or cultural and linguistic diversity, remains an issue in the PPI context.^19^ While it is crucial to clarify the role of each individual and plan PPI according to available time and resources, it is important to think about multiple spaces of involvement so that people with diverse backgrounds are invited and feel welcome to participate. Third, the resources and training necessary for patients and researchers to engage in PPI. There is a need to conceive innovative training practices (for both researchers and patients) that can better take into account the variety of persons' experiential knowledge, pre‐existing skills and needs for establishing a common understanding between patients, members of the public, and researchers.^20^, ^21^ Fourth, along with outcomes assessment, any research project that includes PPI should be accompanied by an evaluation to determine the experiences of the PPI partners, and determine areas to strengthen or improve (i.e., degree of social diversity or the level of shared decision‐making at specific stages of research).^22^, ^23^ Furthermore, although tools such as the Guidance for Reporting Involvement of Patients and the Public (GRIPP2)^24^ aim to facilitate systematic reporting in health and social care research, inconsistent reporting on PPI makes it difficult for the research community to assess and understand the different PPI activities, losing opportunities to learn from previous experiences.

There has been growing interest among decision makers, researchers, and patients to engage PPI work in this constantly evolving cancer care and treatment space. In the field of cancer research, two systematic reviews from 2008^25^ and 2018^2^ explore the use of PPI among people with cancer. Both studies note the increasing recognition of PPI in cancer research and explore specifically why and how patients are involved. Hubbard and colleagues^25^ outline the need for infrastructure to support PPI, including formal recruitment and training to support involvement activities. Furthermore, Hoffmann Pii and colleagues^2^ identified a lack of evidence related to the processes and reporting of PPI, specifically during the definition and prioritization of research topics and the development of recruitment strategies. Since this last review has been published in 2018, there has been significant expansion of research using PPI approaches, and we sought to understand the current state of the literature.

Given the increasing focus on PPI in cancer research, the aim of this review is to: (i) examine the current use of PPI in cancer research and (ii) identify key elements related to PPI and describe their application in cancer research.

## 2 METHODS

To understand the current use of PPI in cancer research and identify how it is being applied in cancer research, we conducted a scoping review using the framework recommended by Arksey and O′Malley^26^ and revised by Levac et al.^27^ The framework consists of six steps: (1) identifying the research question; (2) identifying relevant studies; (3) developing eligibility criteria for study selection; (4) charting the extracted data; and (5) collating, summarizing, and reporting the results; and (6) stakeholder consultation.

### 2.1 Research question

The aim of this scoping review was to review the published scientific literature in PPI in cancer research from 2005 to March 2022 and report specifically on the application and reporting of PPI among these publications. The research questions driving this review are:

1. What is the breadth and depth of scientific literature on PPI in cancer research?
2. How is PPI applied and reported in cancer research?

### 2.2 Identifying relevant studies

We collaborated with a health sciences librarian (UE) experienced in PPI research. Two notable related publications on PPI in cancer were consulted in the development of our search strategy: (1) A 2018 Danish publication^2^ covering literature published between December 2006–April 2017; and (2) A 2008 UK review of the literature^25^ which included searches from 1994–2004. Based on the search parameters and design of these two reviews we set our search dates as 2005 onward to include all recent literature, while also capturing studies that may have been missed in prior publications.

**FIGURE 1:**
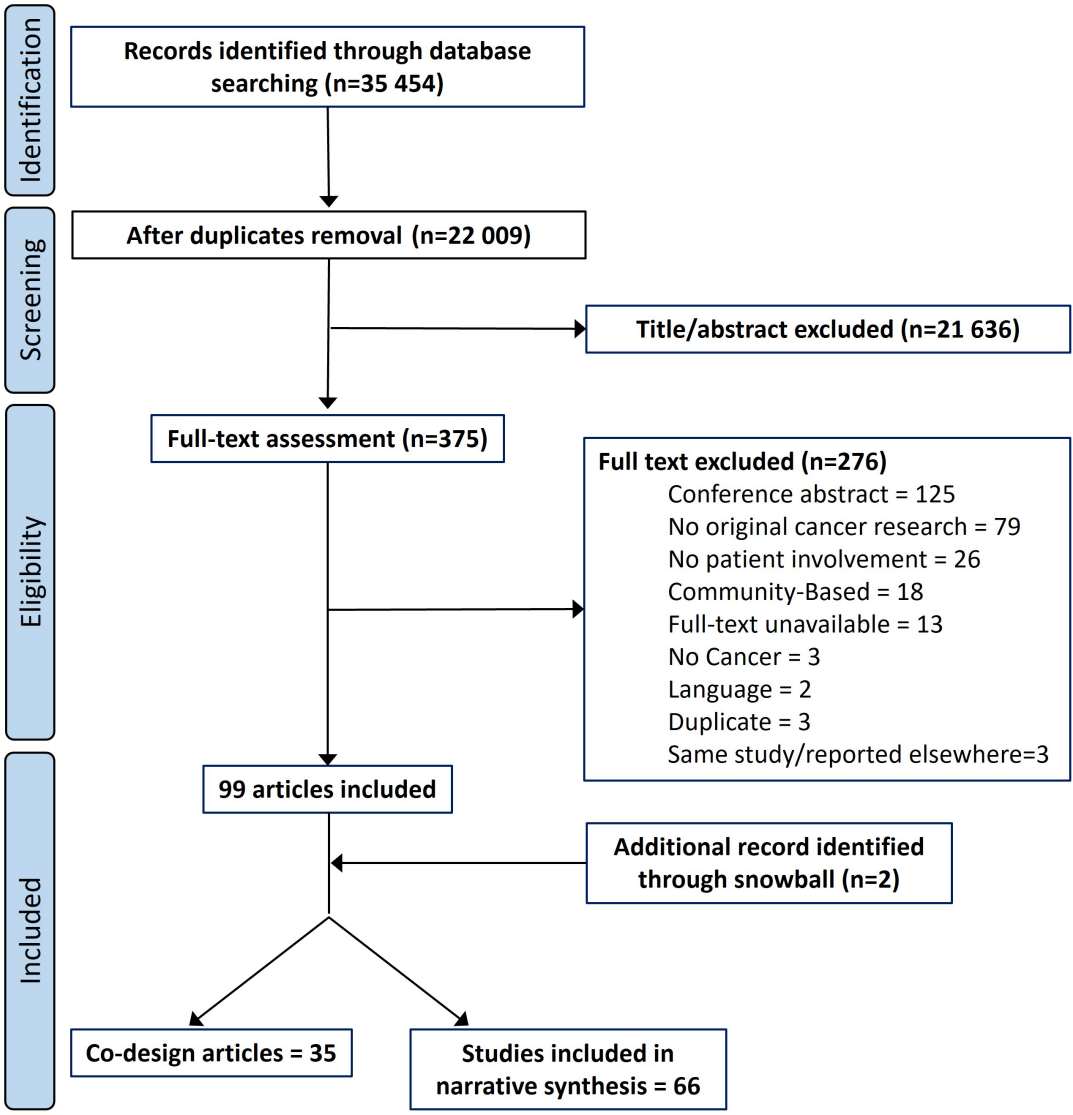
PRISMA flow diagram of the search and selection process.

A search strategy (Table SS1, *) was developed by UE and adapted to the following four databases, chosen as information sources for this scoping review: MEDLINE (OVID interface), Embase (OVID interface), Cumulative Index to Nursing and Allied Health Literature (CINAHL; EBSCO interface), and PsycInfo (EBSCO interface). The four databases were initially searched on March 12, 2021, with an update run March 3, 2022. Additional relevant articles were identified using a snowballing approach. The PRISMA flow diagram of the search and selection process is depicted in Figure 1.

### 2.3 Screening and eligibility criteria

Regarding the screening phase, peer‐reviewed original cancer studies describing the involvement of people affected by cancer (patients and/or relatives) or lay public were considered. There were no restrictions imposed on language within the search strategy; however, only articles that could be effectively translated into English, French, or Spanish were included in this review. We excluded opinion articles, discussion papers, editorials, commentaries, brief reports, protocols, and reviews.

In the eligibility phase (title/abstract screening), the inclusion of articles was restricted to the explicit mention of PPI or related terms. Papers using a Community‐Based Participatory Research approach (CBPR) were excluded. Although sharing similarities with PPI, CBPR approaches traditionally focus on behavioral or social change or inequalities and on community identity rather than individuals or small groups having a living experience with the disease.^28^

### 2.4 Study selection

Studies were exported from the database interface and uploaded into Covidence software.^29^ Following de‐duplication within Covidence, titles and abstracts were screened independently by two members of the review team (MS, SCL, or KRH). Discrepancies in eligibility screening were resolved by discussion to reach consensus. After title and abstract screening, full texts were reviewed (MS, SCL, KRH) for their eligibility. If two authors deemed a full‐text article to be ineligible for this review, the reason(s) were documented.^30^

### 2.5 Charting the extracted data

An extraction grid was developed reflecting the elements related to the research question. The first and last authors piloted the extraction grid with three articles. Relevant data were collected in a tabular form including author, year, country, name of the study, type of disease, characteristics of the participants, characteristics of patients/relatives/public involved, stage of research where PPI was applied, level of involvement, role type, method for involvement, aim for applying PPI, and training and costs associated with PPI. Data extraction was completed independently by two reviewers (SCL and MS). Weekly meetings were conducted to discuss and resolve disagreements and assure reliability during the extraction process. Data were compiled in Microsoft Excel.

Articles describing the use of a co‐design approach (e.g., Experience‐Based Co‐Design, User Centered Design of digital applications) were kept in a separate group as these methodologies often imply active involvement as part of the participatory approach without the stated PPI aim. We identified co‐design studies where patients or members of the public were involved independent of the methodology (e.g., board members, steering committee).

### 2.6 Collating, summarizing, and reporting the results

Results were summarized using a descriptive and a narrative synthesis process. Descriptive results (frequency and percentages) are reported for countries, number of publications per year, methods, participants' characteristics, and cancer type. An iterative process was used to identify categories related to the purpose of PPI in cancer research, the level of involvement and role types, and the different stages of the research process. During the data abstraction phase, we categorized the levels of involvement based on established PPI frameworks (see Table S2) as participation, consultation, collaboration, or partnership. Next, we identified different role types of involvement, which we characterized as personal engagement, advisor/expert, or co‐researcher. Levels and role types were ultimately refined based on the iterative analysis. Definitions for each category are provided in the results section and in Table S5.

### 2.7 Stakeholder consultation

A consultation meeting was held including the research team and two patient partners (diverse in age and cancer experience) with experience in participating in research projects. The meeting aimed to gather input and refine the findings to increase the relevance of the results and confirm their validity in the specific context of PPI in cancer research. Preliminary results were presented by the first and last authors followed by an open discussion on the relevance of these results for the field, any important aspect missing, and how these findings might be disseminated to the stakeholders in the field. The main aspects of the discussion were collected, analyzed, and integrated in the manuscript.

## 3 FINDINGS

In total, 101 articles are included in this review: 66 cancer research studies applying PPI and 35 studies using a co‐design method (Figure 1). We first present an analysis of the 66 papers using a PPI approach, followed by a brief analysis of the 35 studies using a co‐design method.

### 3.1 Characteristics of the studies

**FIGURE 2:**
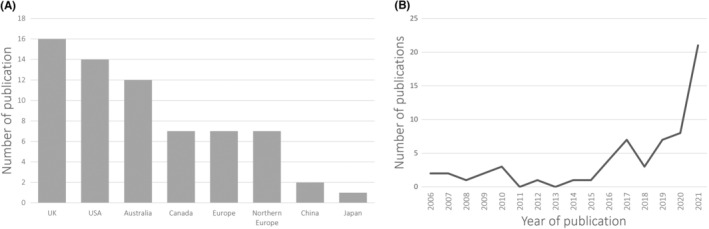
Characteristics of the selected studies based on country (A) and year (B) of publication.

Out of the 66 PPI articles, 16 originated in the United Kingdom,^31^, ^32^, ^33^, ^34^, ^35^, ^36^, ^37^, ^38^, ^39^, ^40^, ^41^, ^42^, ^43^, ^44^, ^45^, ^46^ 14 in the United States,^47^, ^48^, ^49^, ^50^, ^51^, ^52^, ^53^, ^54^, ^55^, ^56^, ^57^, ^58^, ^59^, ^60^ 12 in Australia,^61^, ^62^, ^63^, ^64^, ^65^, ^66^, ^67^, ^68^, ^69^, ^70^, ^71^, ^72^ 7 in Canada,^73^, ^74^, ^75^, ^76^, ^77^, ^78^, ^79^ 2 in China,^80^, ^81^ and 1 in Japan.^82^ European countries included: Switzerland (*n* = 2),^83^, ^84^ The Netherlands (*n* = 2),^85^, ^86^ and Spain (*n* = 1),^87^ as well as two studies comprising several countries (*n* = 2).^88^, ^89^ Northern European countries included Norway (*n* = 3),^90^, ^91^, ^92^Denmark (*n* = 2),^93^, ^94^ Sweden (*n* = 2)^95^, ^96^ (Figure 2A). Overall, an increase in cancer research articles stating the involvement of patients or members of the public is observed from 2015 onwards with a sharp increase noted in 2020 (Figure 2B).

### 3.2 Characteristics of the Patient/Public involved in the studies

**FIGURE 3:**
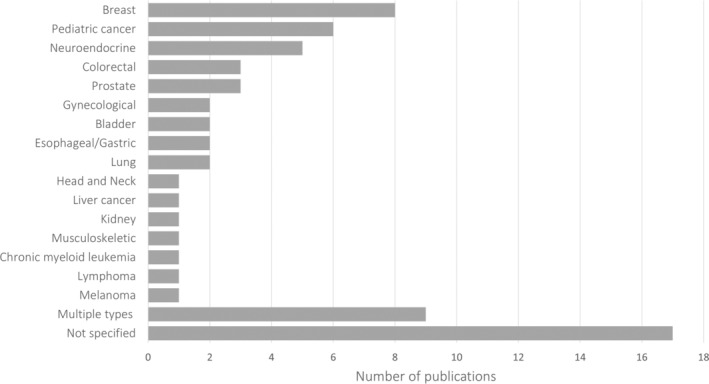
Selected studies distributed based on the main cancer type.

Characteristics of participants involved in the research were extracted. Breast cancer was the type of cancer with the most articles reporting PPI (8/66), followed by pediatric (*n* = 6) and neuroendocrine cancers (*n* = 5). Most of the studies (17/66) did not specify the type of cancer of patients involved or included multiple types (9/66). Regarding the PPI target population, 4/66 included adolescents or young adults (AYAs)^41^, ^44^, ^68^, ^94^ and the rest consisted in adult population, of which 5/62 studies included parents of pediatric patients,^40^, ^53^, ^79^, ^95^, ^96^ 2/62 older adults,^32^, ^76^ and 1 study included patients with intellectual disability^90^ (Table S3). People previously diagnosed with cancer not currently undergoing treatment, including survivors, were the most frequent type of PPI participants (39/66), followed by relatives or informal caregivers (36/66). Furthermore, 6 out of 66 studies incorporated the views of the public (those without cancer or caregiving experience) into cancer research (Table S3 and Figure 3).^32^, ^34^, ^42^, ^58^, ^90^, ^93^

Half of the articles (35/66) included a mention to the PPI population diversity. Diversity referred to age, income, social status, rural/urban area, education, ethnicity, or under‐researched communities (Table S3). Based on the collected data, purposeful sampling was found to be the most commonly used method to ensure diversity among patient partners and members of the public. Various recruitment strategies were employed including hospital consultations, community organizations, charities, recruitment agencies, and even public spaces. Determining the exact number of patients, representatives, informal caregivers, or members of the public involved in each study was difficult, as many articles remained vague when reporting this information. Involvement ranged from one representative in the study steering committee to hundreds in the case of topic prioritization surveys (Table S3).

### 3.3 Purpose of PPI in cancer research

We identified four main purposes for PPI in cancer research (Table S4): (1) to assure that the research is relevant (e.g., defining research priorities or research questions); (2) to assure that the research is appropriate and the research documents are comprehensive (e.g., creating, defining or revising content elements in questionnaires and surveys, study documents, resources, electronic applications' interface); (3) to assure that the research is acceptable, feasible, or attainable (e.g., defining objectives, revising methods, helping and assuring recruitment) and (4) to assure actionability (e.g., defining strategies, next phases, implementation).

### 3.4 Methods used for PPI

**FIGURE 4:**
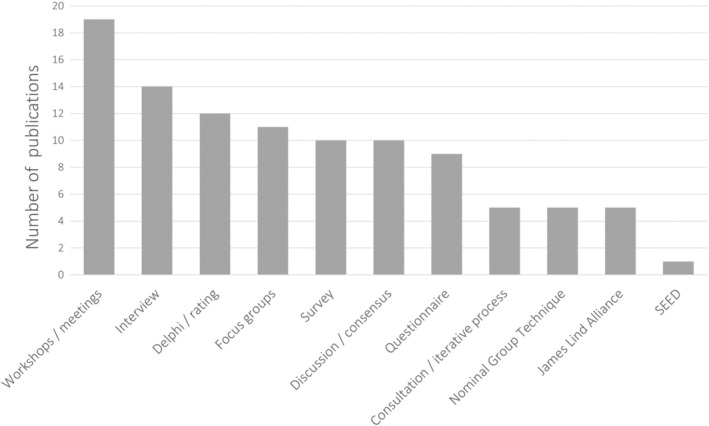
Overview on the different methods applied in PPI approaches. SEED (Stakeholder Engagement in quEstion Development and Prioritization).

The studies included a variety of qualitative and quantitative methods (Figure 4). Workshops and meetings (including steering/advisory board meetings) were the most frequently used methods followed by interviews. In order to reach consensus (i.e., prioritization) or establish ratings, several methods are used ranging from iterative processes (i.e., Delphi method), Nominal Group Techniques, to established methodologies such the James Lind Alliance process^42^, ^44^, ^77^, ^78^, ^79^ or the Stakeholder Engagement in quEstion Development and Prioritization (SEED).^51^

### 3.5 Levels, role types, and research stages of PPI

Based on the continuum of involvement in PPI frameworks (Table S2), we identified four **levels of involvement**. These levels include: (i) Information/Participation: to obtain broad information, opinions, experience, concerning a one‐time or specific task question, or topic (i.e., for identification or validation of a topic via a survey); (ii) Consultation: to obtain feedback and advice on a defined research question or research activity (i.e., revise study documents, content relevance, ratings). Patients or the public take an active role in the research project; (iii) Collaboration: to work directly with patients throughout or at different moments of the research process to ensure that their expectations and concerns are understood and addressed; (iv) Partnership: to establish an equal and active co‐leadership between the patient and the researcher where decisions about the research process are shared (i.e., members of steering committee or study board).

**FIGURE 5:**
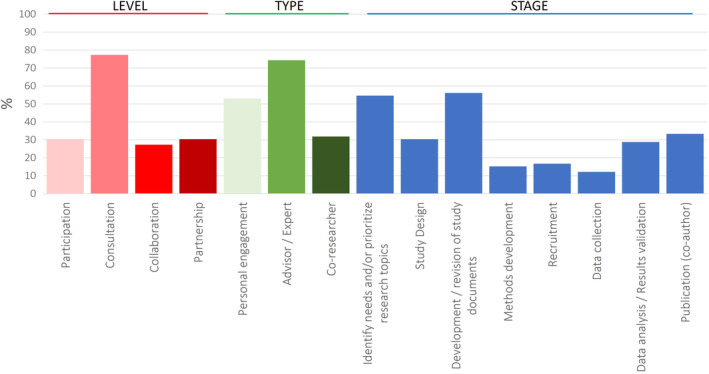
Overview on the level of involvement, role type, and stage of research where PPI is applied. Results are provided in percentages. Definitions and detailed information per study is provided in Suppl. Table 5.

Similarly, we identified three different **role types**, including: (a) Personal engagement: when patients provided a personal perspective and feedback based on their direct experience. This might include members of the public (no affected by cancer); (b) Advisor or Expert: when patients provide advice and guidance from the perspective of both individual and collective experience, bringing the views of a diverse range of patients. For instance, patients participate on associations or organizational boards and hold high‐level of expertise across a broad range of cancer care (i.e., patient representatives or advocates); (c) Co‐researcher: patient was considered as equal partner with essential knowledge necessary for a meaningful contribution to the research project. Involvement in the research process was categorized in 8 **stages** ranging from the identification or prioritization of research topics to publication of the results (Figure 5 and Table S5).

*Consultation* and *Advisor/Expert* were the level of involvement and role most currently used in 77% and 74% of the studies respectively. Almost half of the studies (45%; 30/66) included two or more levels of involvement, and 48% combined two or more roles. PPI was mostly applied during the first stages of research including *prioritization of research topics* (55%) and *development* and/or *revision of study documents* (56%). PPI approaches were less used in the *Recruitment* and *Data collection* stages of research. One third of included studies had patients as co‐authors. A closer look into the data indicates a trend toward a wider PPI uptake encompassing more stages of the research process in recent years (Table S5).

### 3.6 Training, costs, and reporting

Costs related to PPI were mentioned in 38% (25/66) of the studies and referred to whether they paid participants or not, gave a stipend, vouchers/gift cards, covered lunch or travel costs, or were costs relative to the study (e.g., room rental, beverages). In four studies,^31^, ^43^, ^54^, ^87^ training was provided to the patients to collect data and conduct analysis or to co‐facilitate focus groups (Table S3). Regarding systematic reporting, three articles out of the 49 published from 2017 or later used the GRIPP2 tool^46^, ^56^, ^93^ and two others mentioned it as reference.^36^, ^54^

### 3.7 Co‐design studies

In this review, co‐design studies were treated separately as these approaches involve patients as study subjects by collecting their own experience in a first step and to actively working together with healthcare professionals to ultimately improve care. Out of 35 studies identified using a co‐design approach, only 12 stated that they included patients or members of the public in a role other than study participant. More specifically, seven of those 12 studies involved patients in study committee or as co‐researcher, four involved patients in the design of the study, and two in the data collection process (Table S6).

## 4 DISCUSSION

In this paper, we report the findings of an extensive review of the literature to understand the current use of PPI in cancer research and identify how it is being applied. The most salient finding of our review is that involvement of patients and the public in cancer research has increased since 2015 with a burst of publications occurring from 2020 onwards. The UK, United States, Australia, and Canada have been publishing the majority of work in this area. We identified, combined, and synthesized similar key PPI elements across the studies that helped to reframe the purpose, level, role types, and stages of patient involvement in cancer research. Our review exposes several challenges regarding PPI, including the lack of diversity and costs associated with PPI approaches. The expansion of PPI in cancer research points to the need for additional scrutiny on the nature of published PPI.

Results from our review indicate that women with breast cancer remains the population most involved in cancer research. This is not surprising given the highly engaged breast cancer community.^97^ The literature shows that people affected by cancer, particularly women with breast cancer, have been involved in a range of research programs, projects and initiatives especially in the USA, UK, Canada, and Australia.^2^, ^25^ Concern toward the lack of diversity or the presence of overrepresentation of privileged people remains one of the barriers when applying PPI.^98^, ^99^ Several studies have shown that PPI projects involve a majority of persons with higher education and socioeconomic background, while people with learning difficulties, older people, or people from minority ethnic groups are underrepresented.^9^ This underrepresentation in PPI activities by groups with distinct needs warrants reflection, particularly considering the aim of most PPI research to enhance the applicability of the work to the target population. We can echo this concern from our review, wherein only four studies focused on the specific needs of the adolescent/young adults, two on older adults, and one on individuals with intellectual disabilities. Nevertheless, half of the studies mention the characteristics of the PPI participants, acknowledging the awareness for diversity.

Levels of involvement ranged from participation to partnership (from a passive to a higher level of involvement) and role types from personal engagement to co‐researcher (sharing an individual experience to essential knowledge for decision‐making). PPI was mostly used at the level of “consultation” to obtain advice or feedback. “Advisor/Expert,” providing guidance from the perspective of both individual and collective experience, was the role type of involvement most applied. Interestingly, half of the studies included two or more levels and role types of involvement reflecting the engagement in research as part of a continuum as described by several models.^100^, ^101^

Similarly to McCarron et al,^102^ who analyzed the engagement practices of patient partners in health research, we also found that workshops and meetings were the methods used most often followed by interviews. Our observation supports what Greenhalgh^9^ and Langley^103^ describe as a shift toward practical workshops in knowledge creation or “collective making” rather than static “one‐size fits all” frameworks or linear models of research production. This tendency might also explain the difficulty to describe PPI in research dissociated from co‐development, co‐design, or participatory approaches, as we can observe more often that patient and members of the public are involved in a range of different activities, stages, roles, and levels along the same research project.

Furthermore, similar to previous reports,^1^, ^2^, ^104^, ^105^, ^106^ we also observed that PPI practices in cancer research tend to concentrate on the early phase of research to prioritize or design research agendas, while involvement in data analysis and dissemination is uncommon. These observations may point to concerns around an absence of support to fully adopt a PPI approach and whereby PPI is implemented in the “easiest” way possible, leading to the risk of tokenism. Nevertheless, an overview on the involvement at the different stages of research across studies indicates a trend to extend PPI to later stages as seen from studies published after 2020. This escalation coincides with the increase of publications applying PPI approaches observed around this period.

The lack of PPI implementation may also point to patient and researcher needs for greater resources and support. A recent review of the literature on barriers and enablers identified both financial compensation and resources, and training as key factors in PPI.^107^ Interestingly, only one third of papers in our review (38%) included mention of costs associated with the PPI‐related efforts (remuneration, stipends, travel costs, etc.). In addition, only four papers out of the 66, mentioned patients receiving training. Given the complex demands of applying PPI in research, and the expert knowledge of researchers, providing training for patients may allow them to fully understand their role and may begin to address the power differential between researcher and patient.

A final point of reflection relates to the evaluation and reporting of PPI in cancer research. To date, there is still a gap in understanding about PPI's impact on research outcomes and how it is achieved.^108^ On their review, Bird and colleagues aimed to understand this impact.^109^ From the 14 studies they identified, the impact of partnership was mainly evaluated by the research team itself or together with patient partners or other stakeholders, and only two included external reviewers. The authors highlight the urgent need of standardization and transparency when applying a PPI approach to avoid ambiguity in definitions, methods, and reporting of results.^109^ Researchers might consider undertaking initial, midterm, and end‐of‐project evaluations of evolving relationships to increase their understanding of patient involvement.^110^ Furthermore, our results show that systematic and standardized reporting of PPI approaches remain limited. Some efforts have been made in this direction to develop tools to evaluate^111^, ^112^ or report the impact of PPI^24^ on their study. However, standardization in reporting guided by static checklists (e.g., GRIPP2) can also lead to certain limitations or constraints given the broad spectrum of PPI approaches.^113^

## 5 STRENGTHS AND LIMITATIONS

This study has several limitations and notable strengths. The inclusion of studies was restricted to mention of PPI in titles and abstracts or stated methodology from the first articles selection (title/abstract). However, this may have resulted in missed papers. We did not search the gray literature, given our goal of understanding the current deployment of PPI in published cancer research. A final limitation relates to the inclusion of details related to PPI in written reports. We relied upon the authors' accounts of PPI, and it is possible that not all PPI endeavors were included in the manuscripts when they did involve PPI. This study also has notable strengths that include the comprehensive nature of the search and analysis, the extensive data visualization, and both tabular and narrative synthesis. Together these strengths create an important output and resource material to guide future PPI in cancer research.

## 6 CONCLUSION AND RECOMMENDATIONS

This paper serves as a map to illustrate where we are in the implementation of PPI in cancer research. We note the increasing volume of cancer research using a PPI approach, alongside distinct patterns in the role type of involvements. However, we also note limitations in how PPI is being implemented and reported. We recommend that to enhance future cancer research using a PPI approach, authors should report in a reflexive and precise way how and at which level PPI was employed. This may be guided (but not limited to) the use of standardized checklists such as the GRIPP2. Based on our work, we also argue that there is a need for increased evaluation of PPI approaches in cancer research. This may require that publications using PPI are given additional space to justify and explain PPI considering article length restrictions.

## AUTHOR CONTRIBUTIONS

**Sara Colomer‐Lahiguera:** Conceptualization (lead); data curation (lead); formal analysis (lead); funding acquisition (equal); investigation (lead); methodology (lead); project administration (lead); supervision (lead); validation (lead); visualization (lead); writing – original draft (lead); writing – review and editing (lead). **Matthieu Steimer:** Data curation (equal); formal analysis (equal); validation (equal); writing – original draft (equal); writing – review and editing (equal). **Ursula Ellis:** Data curation (equal); methodology (equal); resources (equal); writing – original draft (equal); writing – review and editing (equal). **Manuela Eicher:** Conceptualization (equal); funding acquisition (lead); resources (equal); validation (equal); writing – original draft (equal); writing – review and editing (equal). **Margaret Tompson:** Validation (equal); visualization (equal); writing – original draft (equal); writing – review and editing (equal). **Tourane Corbière:** Validation (equal); visualization (equal); writing – original draft (equal); writing – review and editing (equal). **Kristen Haase:** Conceptualization (equal); data curation (equal); formal analysis (equal); funding acquisition (equal); methodology (equal); resources (equal); software (equal); supervision (equal); validation (equal); visualization (equal); writing – original draft (equal); writing – review and editing (equal).

## FUNDING STATEMENT

Part of this work has been supported by the Foundation BRYN TURNER‐SAMUELS.

## CONFLICT OF INTEREST STATEMENT

The authors declare no conflict of interest.

## ETHICS APPROVAL STATEMENT

Not applicable.

## Supporting information


**Table S1.** Search strategies
**Table S2.** Established frameworks and their different levels of involvement
**Table S3.** Characteristics of PPI participants in cancer research studies
**Table S4.** Purpose of PPI
**Table S5.** Description of the level, type and stages of research where PPI was applied per study
**Table S6.** Co‐design studies identified

## Data Availability

The data that support the findings of this study are available on request from the corresponding author.

## Endnote

* The complete strategy can also be found in https://osf.io/audqy/?view_only=e66742b5b89e4a22a815adf489c2037b
