# Supplementary material for: Patient and public involvement in cancer research: A scoping review
Source: Cancer Med. 2023 Jun 16;12(14):15530–43. doi: 10.1002/cam4.6200 (PMC10417078; doi:10.1002/cam4.6200)
Supplement: Supplementary file 1 — Table S1. Search strategies Table S2. Established frameworks and their different levels of involvement Table S3. Characteristics of PPI participants in cancer research studies Table S4. Purpose of PPI Table S5. Description of the level, type and stages of research where PPI was applied per study Table S6. Co‐design studies identified [file CAM4-12-15530-s001.pdf]

Patient and public involvement in cancer research: a scoping review

SUPPLEMENTARY INFORMATION

Supplementary Table 1. Search strategies

Search strategies also available at: https://osf.io/audqy/?view_only=e66742b5b89e4a22a815adf489c2037b

Database(s): Ovid MEDLINE(R) and Epub Ahead of Print, In-Process, In-Data-Review & Other Non-Indexed Citations, Daily and Versions(R) 1946 to March 11, 2021

1	Patient Participation/	26785
2	patient oriented research.mp.	328
3	patient orientated research.mp.	7
4	patient participation.mp.	28504
5	"patient and public involvement".mp.	826
6	(patient* adj2 (involvement or engag* or collaborat* or led or driven)).mp.	26954
7	(consumer* adj2 (participation or involvement or engag* or collaborat* or led or driven)).mp.	2100
8	(lay adj2 (participation or involvement or engag* or collaborat* or led or driven)).mp.	273
9	(people adj2 (participation or involvement or engag* or collaborat* or led or driven)).mp.	3278
10	(user* adj2 (participation or involvement or engag* or collaborat* or led or driven)).mp.	3489
11	(stakeholder* adj2 (participation or involvement or engag* or collaborat* or led or driven)).mp.	5931
12	(parent* adj2 (participation or involvement or engag* or collaborat* or led or driven)).mp.	5503
13	(caregiver* adj2 (participation or involvement or engag* or collaborat* or led or driven)).mp.	1114
14	(carer* adj2 (participation or involvement or engag* or collaborat* or led or driven)).mp.	386
15	(famil* adj2 (participation or involvement or engag* or collaborat* or led or driven)).mp.	6799
16	((community adj2 (engag* or collaborat*)) and research).ti,ab.	3060
17	(coproduc* or co-produc*).mp.	5183
18	(co-research* or coresearch*).mp.	348
19	(co-design* or codesign*).mp.	1429
20	(patient advisor* and research).mp.	111
21	(advisory council* and research).mp.	320
22	(advisory panel* and research).mp.	497
23	(peer research* or peer-led research).mp.	145
24	(partnership* adj2 research*).mp.	1754
25	patient partner*.mp.	561
26	community partner*.mp.	2854
27	participatory research.mp.	7306
28	action research.mp.	4507
29	(patient-centered adj3 research).mp.	1012

30	(patient-centred adj3 research).mp.	112
31	participatory design.mp.	580
32	(GRIPP or GRIPP2).mp.	107
33	research priorit*.mp.	5832
34	(patient* views and research).mp.	1209
35	or/1-34	108408
36	exp Neoplasms/	3428595
37	(cancer* or neoplasm* or oncolog*).mp.	3510244
38	36 or 37	4058595
39	35 and 38	13034
40	limit 39 to yr="2005 -Current"	9599

Search update run in Ovid MEDLINE March 3, 2022. Added these lines to search:

41. (2021031* OR 202104* OR 202105* OR 202106* OR 202107* OR 202108* OR 202109* OR 20211* OR 2022*).dt,ez,da.
42. 40 AND 42
Search update yielded 1663 results.

Database(s): Ovid Embase 1974 to 2021 March 11

1	Patient Participation/	28827
2	patient oriented research.tw.	332
3	patient orientated research.tw.	9
4	patient participation.tw.	3152
5	patient involvement.tw.	3433
6	"patient and public involvement".tw.	829
7	(patient* adj2 (engag* or collaborat* or led or driven)).tw.	23899
8	(consumer* adj2 (participation or involvement or engag* or collaborat* or led or driven)).tw.	2473
9	(lay adj2 (participation or involvement or engag* or collaborat* or led or driven)).tw.	322
10	(people adj2 (participation or involvement or engag* or collaborat* or led or driven)).tw.	4257
11	(user* adj2 (participation or involvement or engag* or collaborat* or led or driven)).tw.	4017
12	(stakeholder* adj2 (participation or involvement or engag* or collaborat* or led or driven)).tw.	6012
13	(parent* adj2 (participation or involvement or engag* or collaborat* or led or driven)).tw.	6707
14	(caregiver* adj2 (participation or involvement or engag* or collaborat* or led or driven)).tw.	1581
15	(carer* adj2 (participation or involvement or engag* or collaborat* or led or driven)).tw.	540
16	(famil* adj2 (participation or involvement or engag* or collaborat* or led or driven)).tw.	9068
17	((community adj2 (engag* or collaborat*)) and research).tw.	3822
18	(coproduc* or co-produc*).tw.	5865
19	(co-research* or coresearch*).tw.	455
20	(co-design* or codesign*).tw.	1741
21	(patient advisor* and research).tw.	155
22	(advisory council* and research).tw.	304

23	(advisory panel* and research).tw.	339
24	(peer research* or peer-led research).tw.	209
25	(partnership* adj2 research*).tw.	2000
26	patient partner*.tw.	895
27	community partner*.tw.	3560
28	participatory research.tw.	4262
29	action research.tw.	5250
30	(patient-centered adj3 research).tw.	1181
31	(patient-centred adj3 research).tw.	149
32	participatory design.tw.	548
33	(GRIPP or GRIPP2).tw.	54
34	research priorit*.tw.	6785
35	(patient* views and research).tw.	716
36	or/1-35	117804
37	exp *Neoplasm/	3455858
38	(cancer* or neoplasm* or oncolog*).tw.	2882795
39	37 or 38	4530459
40	36 and 39	13298
41	limit 40 to yr="2005 -Current"	11851

Search update run in Ovid Embase March 3, 2022. Added this line to search:

42. limit 40 to dc=20210312-20220303
Search update yielded 2464 results.

Database: EBSCO CINAHL, initial search March 12, 2021:

S1	(MH "Consumer Participation")	20,967
S2	"patient oriented research"	115
S3	"patient orientated research"	1
S4	"patient participation"	1,518
S5	"patient and public involvement"	433
S6	(patient* n2 (involvement or engag* or collaborat* or led or driven))	18,137
S7	(consumer* n2 (participation or involvement or engag* or collaborat* or led or driven))	22,163
S8	(lay n2 (participation or involvement or engag* or collaborat* or led or driven))	248
S9	(people n2 (participation or involvement or engag* or collaborat* or led or driven))	3,945
S10	(user* n2 (participation or involvement or engag* or collaborat* or led or driven))	3,236
S11	(stakeholder* n2 (participation or involvement or engag* or collaborat* or led or driven))	4,191
S12	(parent* n2 (participation or involvement or engag* or collaborat* or led or driven))	5,151
S13	(caregiver* n2 (participation or involvement or engag* or collaborat* or led or driven))	1,229
S14	(carer* (participation or involvement or engag* or collaborat* or led or driven))	944
S15	(famil* n2 (participation or involvement or engag* or collaborat* or led or driven))	6,499
S16	((community n2 (engag* or collaborat*)) and research)	3,098

S17	coproduc* or co-produc*	1,029
S18	co-research* or coresearch*	341
S19	co-design* or codesign*	859
S20	"patient advisor*" and research	39
S21	"advisory council*" and research	144
S22	"advisory panel*" and research	232
S23	"peer research*" or "peer-led research"	113
S24	partnership* n2 research*	1,447
S25	"patient partner*"	329
S26	"community partner*"	2,155
S27	"participatory research"	2,567
S28	"action research"	8,487
S29	"patient-centered" n3 research	601
S30	"patient-centred" n3 research	99
S31	"participatory design"	311
S32	GRIPP or GRIPP2	55
S33	"research priorit*"	5,946
S34	"patient* views" and research	487
S35	S1 OR S2 OR S3 OR S4 OR S5 OR S6 OR S7 OR S8 OR S9 OR S10 OR S11 OR S12 OR S13 OR S14 OR S15 OR S16 OR S17 OR S18 OR S19 OR S20 OR S21 OR S22 OR S23 OR S24 OR S25 OR S26 OR S27 OR S28 OR S29 OR S30 OR S31 OR S32 OR S33 OR S34	81,846
S36	(MH "Neoplasms+")	575,329
S37	cancer* or neoplasm* or oncolog*	662,454
S38	S36 OR S37	734,567
S39	S35 AND S38	6,978
S40	S35 AND S38 [limit applied: 2005-present]	6,114

Search update run in CINAHL March 3, 2022. Added these lines to search:

41. ZD (2021031* OR 202104* OR 202105* OR 202106* OR 202107* OR 202108* OR 202109* OR 20211* OR 2022* OR "in process")
42. S40 AND S41

Search update yielded 1057 results.

Database: EBSCO PsycInfo, initial search March 12, 2021:

S1	DE "Client Participation"	2,409
S2	"patient oriented research"	90
S3	"patient orientated research"	3
S4	"patient participation"	5,817
S5	"patient and public involvement"	249
S6	(patient* n2 (involvement or engag* or collaborat* or led or driven))	9,302
S7	(consumer* n2 (participation or involvement or engag* or collaborat* or led or driven))	5,260

S8	(lay n2 (participation or involvement or engag* or collaborat* or led or driven))	212
S9	(people n2 (participation or involvement or engag* or collaborat* or led or driven))	6,758
S10	(user* n2 (participation or involvement or engag* or collaborat* or led or driven))	3,869
S11	(stakeholder* n2 (participation or involvement or engag* or collaborat* or led or driven))	2,770
S12	(parent* n2 (participation or involvement or engag* or collaborat* or led or driven))	17,496
S13	(caregiver* n2 (participation or involvement or engag* or collaborat* or led or driven))	1,365
S14	(carer* (participation or involvement or engag* or collaborat* or led or driven))	355
S15	(famil* n2 (participation or involvement or engag* or collaborat* or led or driven))	11,550
S16	((community n2 (engag* or collaborat*)) and research)	4,896
S17	coproduc* or co-produc*	1,237
S18	co-research* or coresearch*	1,292
S19	co-design* or codesign*	579
S20	"patient advisor*" and research	33
S21	"advisory council*" and research	158
S22	"advisory panel*" and research	139
S23	"peer research*" or "peer-led research"	144
S24	partnership* n2 research*	1,672
S25	"patient partner*"	195
S26	"community partner*"	2,430
S27	"participatory research"	4,007
S28	"action research"	10,228
S29	"patient-centered" n3 research	904
S30	"patient-centred" n3 research	59
S31	"participatory design"	460
S32	GRIPP or GRIPP2	22
S33	"research priorit*"	1,537
S34	"patient* views" and research	430
S35	S1 OR S2 OR S3 OR S4 OR S5 OR S6 OR S7 OR S8 OR S9 OR S10 OR S11 OR S12 OR S13 OR S14 OR S15 OR S16 OR S17 OR S18 OR S19 OR S20 OR S21 OR S22 OR S23 OR S24 OR S25 OR S26 OR S27 OR S28 OR S29 OR S30 OR S31 OR S32 OR S33 OR S34	84,567
S36	DE "Neoplasms" OR DE "Benign Neoplasms" OR DE "Breast Neoplasms" OR DE "Endocrine Neoplasms" OR DE "Leukemias" OR DE "Melanoma" OR DE "Metastasis" OR DE "Nervous System Neoplasms" OR DE "Terminal Cancer"	53,798
S37	TI cancer* or neoplasm* or oncolog*	31,170
S38	AB cancer* or neoplasm* or oncolog*	51,519
S39	S36 OR S37 OR S38	70,159
S40	S35 AND S39	2,927
S41	S35 AND S39 [search limited to 2005-present]	2,592

Search update run in PsycInfo March 3, 2022.

Reran search and applied limiter: Published Date: 20210101-20221231
Search update yielded 113 results.

Supplementary Table 2: Established frameworks and their different levels of involvement

	Framework	Country (Reference)	LEVELS OF INVOLVEMENT
	The Montreal model<br>CEPPP https://ceppp.ca/en/resources/	Canada (Quebec) (Pomey et al., 2015)	4 levels<br>Information<br>Consultation<br>Collaboration<br>Partnership
	SPOR<br>Strategy for Patient-Oriented Research - Patient Engagement Framework - CIHR (cihr-irsc.gc.ca)<br><br>PORLET<br>https://static1.squarespace.com/static/5c869fd0e666695abe893b3b/t/61b0f04d878a731b75039cdf/1638985805316/PORLET+2021+12+08.pdf	Canada (Amirav et al., 2017)	5 or 6 levels<br>Learn / Inform<br>[Participate]<br>Consult<br>Involve<br>Collaborate<br>Lead/Support/Empower
	INVOLVE NIHR<br>Briefing notes for researchers - public involvement in NHS, health and social care research | NIHR	United Kingdom (National Institute for Health Research (NIHR), 2021)	4 levels<br>Consultation<br>Collaboration<br>co-production<br>user controlled research
	CRUK<br>Patient involvement toolkit for researchers | Cancer Research UK	United Kingdom (Cancer Research UK)	3 levels<br>Participation<br>Engagement<br>Involvement
	National Framework for Consumer Involvement in Cancer Control<br>https://www.canceraustralia.gov.au/sites/default/files/publications/national_consumer_framework_web_504af020f2184.pdf	Australia (Cancer Australia and Cancer Voices Australia, 2011)	5 levels<br>Informing<br>Consulting<br>Involving<br>Partnership<br>Consumer-led
	ONCODE<br>Oncode Institute - Patient Engagement Programme	The Netherlands (Oncode Institute, 2021)	5 levels<br>Informing<br>consulting<br>involving<br>Collaborating<br>Devolving

	KCE<br>Position du KCE concernant l'implication des patients dans les projets de recherche en politique des soins de santé (fgov.be)	Belgium<br>(Cleemput I et al., 2019)	**3 levels**<br>Consultation (targeted or integrated)<br>Collaboration/coproduction<br>Patient-led research
	PCORI<br>The Value of Engagement | PCORI	United States<br>(Frank et al., 2015)	**4 levels**<br>Stakeholder input<br>Consultation<br>Collaboration<br>Shared leadership

REFERENCES

- Amirav, I., Vandall-Walker, V., Rasiah, J., and Saunders, L. (2017). Patient and Researcher Engagement in Health Research: A Parent's Perspective. Pediatrics 140. 10.1542/peds.2016-4127.
- Cancer Australia and Cancer Voices Australia (2011). National Framework, for Consumer Involvement in Cancer Control. Cancer Australia. https://www.canceraustralia.gov.au/sites/default/files/publications/national_consumer_framework_web_504af020f2184.pdf.
- Cancer Research UK. Patient involvement toolkit for researchers. https://www.cancerresearchuk.org/funding-for-researchers/patient-involvement-toolkit-for-researchers.
- Cleemput I, Dauvrin M, Kohn L, Mistiaen P, Christiaens W, and Léonard C (2019). Position of KCE on patient involvement in health care policy research. https://kce.fgov.be/en/position-of-kce-on-patient-involvement-in-health-care-policy-research.
- Frank, L., Forsythe, L., Ellis, L., Schrandt, S., Sheridan, S., Gerson, J., Konopka, K., and Daugherty, S. (2015). Conceptual and practical foundations of patient engagement in research at the patient-centered outcomes research institute. Qual Life Res 24, 1033-1041. 10.1007/s11136-014-0893-3.
- National Institute for Health Research (NIHR) (2021). Briefing notes for researchers - public involvement in NHS, health and social care research. https://www.nihr.ac.uk/documents/briefing-notes-for-researchers-public-involvement-in-nhs-health-and-social-care-research/27371.
- Oncode Institute (2021). Patient Engagement Programme. https://www.oncode.nl/research/programs/patient-engagement-programme.
- Pomey, M.P., Flora, L., Karazivan, P., Dumez, V., Lebel, P., Vanier, M.C., Debarges, B., Clavel, N., and Jouet, E. (2015). [The Montreal model: the challenges of a partnership relationship between patients and healthcare professionals]. Sante Publique 27, S41-50.

Supplementary Table 3. Characteristics of PPI participants in cancer research studies

**Author (year)**	**Country**	**Cancer type**	**PPI participant age /Specificity**	**previous cancer / survivor**	**in treatment**	**palliative/end-of life**	**informal carers / relatives**	**advocates /associations/ representatives**	**public (without direct experience with cancer)**	**Characteristics provided**	Diversity awareness mentioned (Age, Income, Social, rural/urban, Education, Ethnicity, under-research communities)	**Number of PPI participants**	**Training provided**	**Any mention to PPI remuneration / costs**
Kelly 2006	UK	Breast	ADULTS	X							Participants ranged in age from 44 to 61 years of age. The majority of participants were Caucasian (n=9) with one being Afro-Caribbean. Nine were married with one participant widowed.	10 patients		
Wright 2006	UK	Different types	ADULTS	X	X	X	X				Purposively selected population groups: This provides an exploration of the research views and priorities of participants with specific population characteristics often underrepresented in research studies, including people from diverse ethnic minoritized backgrounds (South Asian, Black), specific age groups [teenagers (13–19 years), over 75 years old], and patients in the palliative phase of illness.	25 patients: past patients, carers or people working with patients in a consumer involvement capacity of which 15 became co-researchers	**X**	**X**
Carey 2007	AU	Not specified	ADULTS		X						we ensured that the interviews selected for the resource showed a range of different types of people, in order to maximize audience identification	57 patients		
Corner 2007	UK	Different types	ADULTS	X	X	X	X	X		X	To ensure that people from diverse communities and backgrounds were included, consultation groups were also run with purposively selected participants from frequently under researched communities.	105 patients<br>15 patients co-researchers	**X**	
Juraskova 2008	AU	Breast	ADULTS	X				X			Women were on average 60 years of age and 29 were married. Two women had postgraduate qualifications, and 15 were in professional occupations. All but one woman was born in Australia or the UK, 11 had medical training and 11 had a chronic medical condition, including osteoporosis (n = 3). The sample was representative of women participating in the IBIS-I trial.	31 women participating in a breast cancer prevention trial and who are currently in follow-up and the Australian New Zealand Breast Cancer Trials Group (ANZ BCTG) Consumer Advisory Board		
Head 2009	US	Head and Neck	ADULTS	X								39 past patients involved to give input for the development of the algorithm via a survey		
Saunders 2009	AU	Neuroendocrine	ADULTS		X		X			X		6 patients, 5 caregivers and key stakeholder comprising consumers, representatives of the Cancer Council and researchers		X
Forbes 2010	UK	Breast	ADULTS (women aged 60 to 75)	X	X				X	X	We recruited women with breast cancer with the help of two cancer charities: Macmillan, which advertised for volunteers on its website, and Breast Cancer Care, which sent out a flyer with its newsletter. We also directly contacted an Asian women's breast cancer support group. We recruited women who had not had a previous diagnosis of breast cancer using research recruitment agencies, who approached women in	69 women:<br>15 women had a previous diagnosis of breast cancer and 54 did not		X

											public areas and from existing databases of people willing to take part in research, to invite them to participate.<br>We aimed to achieve a mix of women by age and income and living in rural and urban locations. We achieved a good range of characteristics by age, ethnic group, income, rural or urban residence and living arrangements.			
Robotin 2010	AU	Neuroendocrine	ADULTS	X			X					consumer representatives:<br>patients with a PC diagnosis, current and bereaved carers of patients with PC.		
Skorpen 2010	NO	Not specified	ADULTS (intellectual disability)	X			X		X			19 participants:<br>2 with ID and cancer diagnosis, 10 with ID and experience with cancer, 2 mothers of patients with ID with cancer, 5 caregivers of people with ID		
Saunders 2012	AU	Not specified	ADULTS	X			X	X			Workshop: Participants ranged in age from 18 to 72 years. More females (n=18) than males (n= 14) attended the workshop.<br>Questionnaires: The majority of respondents were female (56%) aged between 61–70 years (41%).	89 participants:<br>32 patients and caregivers (workshop) and 57 via questionnaire		
Boelens 2014	Europe	Colorectal	ADULTS	X				X				5 participants:<br>patients representatives and colon or rectal cancer survivors		
Taylor 2015	UK	Pediatric cancer	AYAS	X							young people aged 17–26 years, eight female, who were diagnosed with cancer aged between 14–25 years	9 young people		
Gerritsen 2016	NL	Neuroendocrine	ADULTS		X	X				X		150 patients diagnosed with pancreatic or periampullary cancer		
Huddy 2016	UK	Esophageal/Gastric	ADULTS					X	X		Limitations: to weigh the benefits and challenges of each and to recruit different participants with different backgrounds and motivations.<br>we hope to have achieved an overall representative sample in the course of the three events, although sociocultural and clinical characteristics of participants were not recorded.	38 participants:<br>members of the Oesophageal Patients Association (n=12) and general public without previous experience of OG cancer (n=26)		X
Sperling 2016	DK	Different types	AYAS	X	X						AYA patients differing in age (range 17–38 years), cancer type (breast cancer, lymphoma, sarcoma, blood cancer, and ovarian cancer), gender, and representing diverse geographical areas.	21 AYAs<br>And a youth panel was involved through the entire process (9 AYAs)		
Wan 2016	UK	Endometrial	ADULTS	X	X		X		X	X	the lay participants predominantly identify as white and under 60 years old. It is notable that individuals of Asian (i.e. people identifying as of Indian, Pakistani, Bangladeshi or other South Asian ethnicity) and Black ethnicity and older women who make up a substantial proportion of women diagnosed with EC are under-represented. In order to ensure that views of older women were represented during the initial consultation phase and the final consensus meeting, we specifically invited several women over 65 years of age to participate over and above those recruited from generic call out.	211 participants:<br>steering group of representatives and 211 patients, carers and individuals who identified themselves as being at risk of EC		
Crawford 2017	US	Not specified	ADULTS	X	X						11 men and 8 women. The racial and ethnic breakdown was 8 African American, 1 Asian American, 7 white, and 3 Latino	37 participants:<br>7 oncology patient partners (paid consultants)<br>19 participants for usability<br>11 patients for feasibility		X

Holch 2017	UK	Different types	ADULTS		X			X		X		22 participants:<br>13 patients<br>9 advocates		
Javid 2017	US	Breast	ADULTS	X							All had completed at least a high school education, and 9/13 had a college degree. The majority (11/13) were non-Hispanic white and 2/13 were Black/African-American. Marital and employment status were heterogeneous with no majority seen for either variable.	16 participants:<br>1 patient advisor advisory panel of relevant stakeholders; 15 patients participated in focus group and questionnaire		
Meads 2017	UK	Not specified	ADULTS			X					40% were male, median age 70 years; age range 48–86 years	10 patients with pain from advanced cancer		
Schneider 2017	CA	Musculoskeletic	ADULTS					X				1 representative in Phase III Expert Panel Consensus (not determined)		
Segelov 2017	CA<br>AU<br>NZ	Neuroendocrine	ADULTS	X			X	X				73 patients (36% of the participants)<br>not determined whether survivors or in treatment.<br>Patient/Advocate Panel included patients, caregivers, patient advocates, support societies, and health care consumer representatives		X
Treiman 2017	US	Colorectal	ADULTS	X	X		X	X			Limitations: sample size for cognitive testing participants was limited in size and geographic diversity.	29 participants:<br>23 patients<br>6 family members/caregivers	X	
Melnychuk 2018	UK	Different types	ADULTS	X	X		X	X			Our sample represented five PPI groups comprising patients (current and former) and carers.<br>Limitation: did not collect information on socioeconomic status in the PPI sub-group or age of any of the research participants	22 participants:<br>3 patient representatives to review the text; 19 patients and caregivers for the consultations survey		
Smith 2018	US	Bladder	ADULTS	X	X			X		X	Another challenge encountered was ensuring diverse representation within the Bladder Cancer Advocacy Network with a high-tech approach. Diverse representation should ideally include all patient populations, including those who are hard to reach. Hard-to-reach patient populations include those with low literacy, barriers to using the Internet, numerous work/family obligations, and other barriers that preclude the time and resources to participate in online surveys.	1388 participants:<br>Patients and patient advocated from the Bladder Cancer Advocacy Network: 354 responded to the research prioritization survey in year 1 and 1034 in year 2)		
Wikman 2018	SE	Pediatric cancer	ADULTS (parents)				X				Limitations: It should also be considered that although [participants] of both genders and from different socioeconomic backgrounds were included, the sample was small and recruited from a small geographical area.<br>Future research may benefit from adopting more assertive recruitment methods to identify researcher partners from the wider community of parents of children previously treated for cancer	6 parents		X
Badia 2019	ES	Different types	ADULTS					X			The eight-member panel was too small to be regarded as representative of all patients.	8 Participants:<br>2 patient representatives of patient associations; 2 of cancer patients associations; 4 of specific cancer type patients associations	X	
Jibb 2019	CA	Not specified	ADULTS					X				1 patient representative		

Mayland 2019	UK (7 countries involved)	Not specified	ADULTS				X	X		X	hospital volunteers or representatives from PPI forums<br><br>ensure that there was male representation; and in addition, some specific sub-groups were targeted within certain countries, for example, Turkish volunteers in Germany.	<u>83 participants:</u><br>48 PPI representatives; 35 bereaved relatives		
Payne 2019	US	Lymphoma	ADULTS	X			X				Phase I: Participants predominantly were female (11 participants; 73%) and included respondents who self-identified as white (7 participants; 46.7%) or black/African American (3 participants; 20%).<br>Phase II: median age was 63 years (range, 21-80 years). Participants predominantly were female (55%), white (17 participants; 89.5%), and lived in rural areas (63.2%), with 1 African-American participant and 1 Hispanic participant identified.<br>Limitations: One limitation of the current study is the lack of diversity with regard to race and sex among the study sample. The majority of participants were white and female, thus limiting generalizability.	<u>34 participants:</u><br>8 lymphoma survivors and 7 lymphoma caregivers for phase I (Focus groups)<br>19 patients/caregivers for phase 2 (individual interviews)		
Phillips 2019	UK	Pediatric cancer	ADULTS (parents and childhood cancer survivor)	X			X					<u>5 participants:</u><br>4 parents;<br>1 survivor		X
Rafie 2019	US	Lung	ADULTS		X		X				Topic Group members were predominantly female (76%) with greater than a high school education (91%). TGs had greater representation from adults older than 65 years (28%) and people of Caucasian race (62%).<br>Participants of varying socioeconomic, educational, and professional backgrounds contributed to the lung cancer outcomes research agenda.<br>The selection process ensured diverse viewpoints on the problem. This is illustrated by the fact that individual representatives within each group fell into five of the seven stakeholder categories outlined in the 7Ps Framework for Stakeholder Engagement, including patients and the public, providers, purchasers, payers, and policy makers.	<u>7 participants:</u><br>lung cancer patients and caregivers		X
Janssen 2020	AU	Breast	ADULTS					X				<u>1 consumer representative</u>		
Nicholas 2020	UK	Esophageal / diferent types	ADULTS	X			X				There was a wide geographical representation. In an attempt to acquire a broader view, we deliberately invited and successfully recruited patients from non-urban areas of the UK and patients with no prior experience in PPI work, giving a voice to an often under-represented group of patients.	<u>21 participants:</u><br>patients and caregivers		X
Birkeland 2020	DE	Prostate	ADULTS	X					X	X	Public sphere selecting men encountered at pedestrian streets, in market malls, in hospitals, and among academic course at tenders with various backgrounds in middle sized provincial towns	<u>33 participants:</u><br>30 men of the public;<br>3 patients		X
Schmidt 2020	CH	Breast	ADULTS	X			X	X				<u>8 Participants:</u><br>4 Breast cancer survivors;<br>1 patient advocate;<br>2 family members/relatives;<br>1 patient partner		
Snaman 2020	US	Pediatric cancer	ADULTS (bereaved parents)				X					<u>5 bereaved parents</u>		

Ector 2021	NL	chronic myeloid leukemia	adults		X					x		203 CML patients treated or starting TKI.		
Handley 2021	US	different types	adults		X		X	X		x	participants from a diverse group of sites across the catchment area, with representation from patients and caregivers engaging in care at the SKCC academic hub, an urban community hospital, and a suburban community hospital.<br><br>In addition, although a variety of cancer types and demographics were represented in both the focus groups and cognitive interviews, the majority of participants were White and female, were relatively young for patients with cancer, and had relatively high income and education, which may have affected the ultimate tool by overemphasizing or underemphasizing certain domains. In our catchment area, approximately 52% of patients are female, 72.6% are Caucasian, and 16.9% are Black, with 63% of patients age older than 65 years.	40 participants:<br>Patient and family advisory committee before starting;<br>23 participants (17 patients and 6 caregivers) across four focus groups;<br>17 participants in cognitive interviews		
Da Silva Lopes 2021	CH	Melanoma	adults					X				1 patient advocate		
Schilstra 2022	AU	different types	adolescent and young adult (AYA)	X						x	The six AYAs were on average 24.2 years old (R = 19–31), 83% (N = 5) female, and all Caucasian-Australian.<br>All but one came from the local Sydney area	6 AYA cancer survivors		X
Taggart 2021	AU	colorectal	adults	X								1 consumer with lived experience of cancer		
Yan 2021	US	Lung	adults				X	X	X		12 PAC members from various racial backgrounds: one-third were women, one was African American and one was Hispanic	12 participants:<br>Veterans and Veteran advocates patient advocates included senior Wisconsin members of the American Cancer Society, American Legion and Vietnam Veterans of America organizations.		X
Mazariego 2020	AU	Not specified	adults	X			X				Participants were from five of eight Australian states and territories with the majority residing in New South Wales and Western Australia	9 participants:<br>5 consumers in Delphi round 1 and 4 in round 2; Working Group: cancer patients and survivors		

Stover 2021	US	different types	adults		X		X	X		X	diverse demographic and clinical characteristics of patients with cancer. Patients were purposively sampled to maximize the variety of responses rather than establish generalizable samples. Goal: to recruit at least 20% of the total patients who were >65 years of age, had an ethnic minority heritage, and/or had a high school education or less.<br>Of the 56 patients interviewed, 48%were women, 34% were age $ 65 years, 23% were ethnic minority, and 20% had a high school education or less. Cancer types included genitourinary (32%), GI (27%), breast (21%), and lung (20%). Primary caregivers (n = 21) were 71% female, 24% age ≥ 65 years, 76% non-Hispanic white, and 14% with a high school education or less. Caregiver relationships were typically spouse/partner or an adult child.	56 patients		
Perry 2021	US	Not specified (metastatic / palliative)	adults			X	X					10 patient participants:<br>Stakeholder advisory board was comprised of patients with metastatic cancer, family members		
Dunn 2021	AU	Prostate	adults	X				X		x	community groups and consumers from diverse backgrounds, including LGBTQIA people and those from regional, rural and urban settings.	16 participants:<br>People with experience in provision of support in the community from a range of professional and academic organizations		
Wang 2021	CN	Liver cancer	adults		X							16 patients with PLC		
Gibson 2021	UK	Not specified	AYA	X	X		X	X				4 representatives as co-authors<br>expert steering group including young people		
Wong 2021	CN	Not specified	adults	X	X		X			X		17 participants:<br>patients with cancer [n=5], cancer survivors [n=6], caregivers [n=6].		
Adams 2021	CA	Not specified	adults									185 participants:<br>4 cancer survivors and support persons in the workshop; 78-55-48 participants in the 3 Delphi rounds		
Sekse 2021	NO	Gynecological	adults	X				X				10 women participants		X
Bergerod 2021	NO	Not specified	adults				X	X		x		5 next-of-kin representatives		X

Haase 2021	CA	Not specified	older adults	X	X		X	X		x	Participants attending from rural and urban areas, thereby representing a more diverse cross-section of viewpoints.	36 participants:<br>1 co-researcher and 35 older adults and caregivers (25 patients, 8 caregivers, 2 both)		X
Woodford 2022	SE	Pediatric	adults (parents)				X					4 Participants:<br>parent research partner (PRP) group consisted of 2 mothers and 2 fathers		
Crawford-Williams 2021	AU	Not specified	adults (survivors)	X				X				18 participants:<br>10 cancer survivors; 8 cancer advocacy representatives		
Zhong 2021	CA	Breast	adults	X			X	X			24 participants face-to-face workshop: varying socioeconomic and cultural backgrounds arising from geographically diverse areas throughout Canada.	552 participants:<br>Steering group included 5 breast reconstruction patients, 2 community partners who were also breast cancer survivors;<br>Survey 521 patients and caregivers;<br>face-to-face workshop 24 participants including breast reconstruction patients, mastectomy patients, family members, caregivers		
Smith 2021	US	Bladder	adults	X			X	X			We also recognize the need to improve socioeconomic and racial/ ethnic diversity among our patient advocates. Identifying engaged patient partners with diverse backgrounds and perspectives is integral to designing a generalizable study that likewise supports a diversity of patients	advocate advisory board (AAB) composed of patients and caregivers and led by a key advocacy organization leader		
Kanapathy 2021	UK	Breast	adults	X			X					85 participants:<br>Patients were part of the steering committee; Delphi panel 12 patients; Survey 71 patients; 2 carers		
Jones 2017	CA	Kidney	adults		X		X					19 participants:<br>Steering group included 7 patients/caregivers<br>12 participants (Workshop: 10 patients; 2 caregivers).		
Beyer 2021	Europe (consortium)	Prostate	adults		X			X				30 participants:<br>3 patient experts for the consensus meetings; 27 patients from different European countries for the interviews		

Furukawa 2022	JP	Not specified	adults	X			X					6 Participants<br>Co-author KH is a cancer patient himself; 3 cancer survivors; 2 bereaved family members.		
Shojaie 2021	US	Endocrine	adults	X	X		X				race, education, religion, health insurance	50 participants:<br>4 patients and family members in stakeholder Advisory panel; 46 patients with medullary thyroid cancer or survivor; 10 family members.		X
Cavers 2020	UK	Not specified	adults		X		X	X			To reach a wide audience, relevant organisations such as the National Institute for Health Research's INVOLVE, Our Voice Scotland, and the Alliance were tagged<br><br>Limitations: There are also limitations to the number of people taking part in the consultation and reflections to be made on the planning process to ensure a broader scope and diversity of consultants, speaking to existing debates around whether or not patient involvement includes the voices of a wide enough group of people, including those from marginalised groups and those less able to expertly self-advocate and articulate their views and beliefs	19 participants:<br>Workshop: 6 attendees and Online consultation: 10 responses<br>1 patient member of the panel member<br>1 patient member as Advisory Board<br>1 patient invited to join the research team	Importance mentioned, but training not provided	
Flegg 2020	CA	Retinoblastoma	Adults (parents or survivors)	X			X			X	Respondents were primarily female (50 [85%]) and Ontario residents (34 [58%]). Patients were parents (28 [74%]) or survivors (10 [26%]); the mean patient age was 38 (standard deviation 8) years.<br><br>Limitations: Patient survey participants may not have been representative of the broader Canadian retinoblastoma community. Only 38 patients participated in the survey, largely female parents from Ontario	53 participants<br>Online survey: 38 patients<br>The working group included 1 patient<br>Steering committee: 4 patients<br>workshop participants:10 patients		X

Supplementary Table 4 : Purpose of PPI

Author	Kelly 2006	Wright 2006	Carey 2007	Corner 2007	Juraskova 2008	Head 2009	Saunders 2009	Forbes 2010	Robotin 2010	Skorpen 2010	Saunders 2012	Boelens 2014	Taylor 2015	Gerritsen 2016	Huddy 2016	Sperling 2016	Wan 2016	Holch 2017	Javid 2017	Jones 2017	Meads 2017	Schneider 2017	Segelov 2017	Treiman 2017	Melnychuk 2018	Smith 2018	Wikman 2018	Badia 2019	Crawford 2019	Jibb 2019	Mayland 2019	Payne 2019	Phillips 2019	Rafie 2019	Birkeland 2020	Cavers 2020	Flegg 2020	Janssen 2020	Mazariego 2020	Nicholas 2020	Schmidt 2020	Snaman 2020	Adams 2021	Beyer, 2021	Bergerod 2021	Crawford-Williams 2021	Da Silva Lopes 2021	Dunn 2021	Ector 2021	Gibson 2021	Haase 2021	Handley 2021	Kanapathy 2021	Perry 2021	Sekse 2021	Shojaie 2021	Smith 2021	Stover 2021	Taggart 2021	Wang 2021	Wong 2021	Yan 2021	Zhong 2021	Furukawa 2022	Schilstra 2022	Woodford 2022	
to assure that the research is **meaningful and relevant** (e.g. defining priorities or research questions)		X		X			X		X		X						X			X		X	X	X		X				X		X		X		X	X		X				X			X		X		X	X		X					X			X	X	X		X		28
to assure that the research is **appropriate** (e.g creating, defining or revising content elements in questionnaires and surveys, study documents, resources, interface)	X	X	X		X	X	X		X	X		X	X	X	X	X		X	X		X			X	X		X	X	X		X	X	X	X	X		X	X			X	X		X	X		X	X				X		X	X	X	X		X	X				X		X	42
to assure that the research is **acceptable, feasible, attainable** (e.g. defining objectives, revising methods, helping with recruitment)			X		X			X			X				X				X							X			X				X		X					X	X			X										X	X		X	X								X	18
to assure **actionability** of the research project (e.g. defining strategies, next phases; implementation)												X																		X			X																																		3

Supplementary Table 5 : Description of the level, type and stages of research where PPI was applied per study

		Kelly 2006	Wright 2006	Carey 2007	Corner 2007	Juraskova 2008	Head 2009	Saunders 2009	Forbes 2010	Robotin 2010	Skorpen 2010	Saunders 2012	Boelens 2014	Taylor 2015	Gerritsen 2016	Huddy 2016	Sperling 2016	Wan 2016	Holch 2017	Javid 2017	Jones, 2017	Meads 2017	Schneider 2017
LEVEL OF INVOLVEMENT	**Participation:** to obtain information, opinions, experience, concerning a specific task (i.e. comprehensibility, interface, usability).			x		x	x				x					x							
	**Consultation:** to obtain feedback and advice on specific research questions or research activities (i.e.revise study documents, content relevance, ratings).	x		x		x		x	x	x	x	x	x	x	x	x	x	x		x		x	x
	**Collaboration:** to work directly with patients throughout the research process to ensure that their expectations and concerns are understood and addressed.		x		x								x				x	x	x	x			
	**Partnership:** to establish an equal and active co-leadership between the patient and the researcher where decisions about the research process are shared.		x		x								x						x	x	x		
ROLE TYPE OF INVOLVEMENT	**Personal engagement:** patient provides a personal perspective and feedback from direct experience. Might include members of the public (no affected by cancer).			x			x	x	x	x	x	x		x		x	x	x					
	**Advisor / Expert:** patient provides advice and guidance from the perspective of both individual and collective experience, bringing the views of a diverse range of patients.	x	x	x	x	x			x			x	x		x	x	x	x	x	x		x	x
	**Co-researcher:** patient is considered as equal partner with essential knowledge necessary for the research project and supports the implementation of PPI in all stages of the research.		x		x								x					x	x	x	x		
STAGE OF INVOLVEMENT	**Identify needs and/or prioritize research topics**	x	x		x			x		x		x				x		x			x		x
	**Study Design**		x		x				x												x		
	**Development / revision of study documents**			x		x	x				x		x	x	x		x		x	x		x	
	**Methods development**		x																		x		
	**Recruitment**																				x		
	**Data collection**		x		x																		
	**Data analysis / Results validation**		x		x													x		x			
	**Publication (co-author)**											x	x			x		x	x				

		Segelov 2017	Treiman 2017	Melnychuk 2018	Smith 2018	Wikman 2018	Badia 2019	Crawford 2019	Jibb 2019	Mayland 2019	Payne 2019	Phillips 2019	Rafie 2019	Birkeland 2020	Cavers 2020	Flegg 2020	Janssen 2020	Mazariego 2020	Nicholas 2020	Schmidt 2020	Snaman 2020	Adams 2021	Beyer, 2021
LEVEL OF INVOLVEMENT	**Participation**		x	x		x				x			x	x	x	x							x
	**Consultation**	x	x	x	x	x	x	x		x	x	x	x	x	x	x	x	x	x	x		x	x
	**Collaboration**		x			x		x	x						x	x		x		x	x		
	**Partnership**		x												x	x				x	x		
ROLE TYPE OF INVOLVEMENT	**Personal engagement**	x	x	x	x	x		x		x	x	x	x	x	x	x				x			x
	**Advisor / Expert**	x	x		x	x	x	x			x	x	x	x	x	x	x	x	x	x	x	x	x
	**Co-researcher**		x						x						x	x				x	x		
STAGE OF INVOLVEMENT	**Identify needs and/or prioritize research topics**	x	x	x	x				x		x	x	x		x	x		x		x		x	
	**Study Design**		x					x				x			x	x			x	x	x		
	**Development / revision of study documents**		x	x		x	x	x		x				x	x	x	x		x	x	x		x
	**Methods development**		x									x									x		
	**Recruitment**		x												x	x				x	x		
	**Data collection**																			x			
	**Data analysis / Results validation**		x												x	x				x	x		
	**Publication (co-author)**		x		x		x		x		x					x		x	x	x			

		Bergerod 2021	Crawford-Williams 2021	Da Silva Lopes 2021	Dunn 2021	Ector 2021	Gibson 2021	Haase 2021	Handley 2021	Kanapathy 2021	Perry 2021	Sekse 2021	Shojaie 2021	Smith 2021	Stover 2021	Taggart 2021	Wang 2021	Wong 2021	Yan 2021	Zhong 2021	Furukawa 2022	Schilstra 2022	Woodford 2022	66 (n)	%
LEVEL OF INVOLVEMENT	**Participation**					x			x	x				x	x		x							20	30
	**Consultation**	x	x	x	x		x	x		x	x		x			x		x			x	x	x	51	77
	**Collaboration**											x		x										18	27
	**Partnership**			x			x	x		x			x	x					x	x	x			20	30
ROLE TYPE OF INVOLVEMENT	**Personal engagement**					x	x			x			x	x	x		x	x				x		35	53
	**Advisor / Expert**	x	x	x	x			x	x	x	x	x		x		x			x		x		x	49	74
	**Co-researcher**			x			x	x		x			x	x						x	x			21	32
STAGE OF INVOLVEMENT	**Identify needs and/or prioritize research topics**	x	x	x			x	x		x				x	x		x	x	x	x		x		36	55
	**Study Design**						x		x	x	x		x	x						x	x			20	30
	**Development / revision of study documents**				x	x	x		x		x	x	x	x		x				x	x		x	37	56
	**Methods development**						x			x			x	x						x				10	15
	**Recruitment**						x			x	x			x						x				11	17
	**Data collection**						x	x						x						x	x			8	12
	**Data analysis / Results validation**	x		x			x	x		x		x	x	x						x	x			19	29
	**Publication (co-author)**			x	x		x	x						x	x					x	x			22	33

Supplementary Table 6: Co-design studies identified

First author	Publication year	Title	Method / approach	Level of implication	
Anderson	2021	THRIVE intervention development: using participatory action research principles to guide a mHealth app-based intervention to improve oncology care.	participatory action research	patients as subjects	participation
Bangerter	2021	A hybrid method of healthcare delivery research and human-centered design to develop technology-enabled support for caregivers of hematopoietic stem cell transplant recipients.	design thinking	patients as subjects	participation
Bessa	2021	Designing a Pragmatic Intervention to Help Improve the Bladder Cancer Patient Experience.	discussion groups	patients as subjects	participation
Brady	2020	Using experience-based co-design to improve the pre-treatment care pathway for people diagnosed with head and neck cancer.	experience-based co-design	patients as subjects	participation
Brady	2022	An evaluation of the provision of oncology rehabilitation services via telemedicine using a participatory design approach.	participatory design	patients as subjects	participation
Brown	2020	Using qualitative and co-design methods to inform the development of an intervention to support and improve physical activity in childhood cancer survivors: a study protocol for BEing Active after ChildhOod caNcer (BEACON).	co-design	patients as subjects	participation
Cooley	2018	Algorithm-based decision support for symptom self-management among adults with Cancer: results of usability testing.	user co-design	patients on expert design committee	
Grant	2021	What do cancer survivors and their health care providers want from a healthy living program? Results from the first round of a co-design project.	user co-design	patients as subjects	participation
Grynne	2022	Integrating perspectives of patients, healthcare professionals, system developers and academics in the co-design of a digital information tool.	experience-based co-design	patients on expert design committee	
Hall	2021	Patient and health care professional co-development of an Acceptance and Commitment Therapy intervention to support hormone therapy decision-making and well-being in women with breast cancer.	co-design	patients as subjects	participation
Houghton	2022	Free Time For Wellness: a co-designed intervention utilizing social networks to encourage physical activity for cancer prevention among low resourced mothers.	co-design	patients as subjects	participation
Huh	2021	Making of Mobile SunSmart: Co-designing a Just-in-Time Sun Protection Intervention for Children and Parents.	co-design	patients on expert design committee	
Hyatt	2020	Co-design and development of online video resources about immunotherapy with patients and their family	experience-based co-design	unclear who is on the steering committee - not explicit	
McGrath	2021	Co-design and implementation of an exercise intervention for women with ovarian cancer	review paper		
McMullen	2018	Designing for impact: identifying stakeholder-driven interventions to support recovery after major cancer surgery.	co-design	patients as subjects	participation
Mehdizadeh	2021	mHealth Self-Management System to Supporting Children with a Acute Lymphocytic Leukemia (ALL) and their caregivers in low-middle income country: Qualitative Co-Design Study.	co-design	patients as subjects	participation

Moser	2021	Improving the experience of older people with colorectal and breast cancer in patient-centred cancer care pathways using experience-based co-design.	experience-based co-design	patients and community representatives included in co-design	
Nguyen	2021	Developing an Online Tool to Promote Safe Sun Behaviors With Young Teenagers as Co-researchers.	co-design	patients as co-researchers	
Perera	2022	Codesigning a supportive online resource for Australian cancer carers: a thematic analysis of informal carers' and healthcare professionals' perspectives about carers' responsibilities and content needs.	co-design	patients as subjects	participation
Perry	2021	iSelf-Help: a co-designed, culturally appropriate, online pain management programme in Aotearoa.	co-design	patients on advisory board	
Petit-Steeghs	2021	Co-creating an empowering health education intervention for urological cancer patients	co-design	patient representative organizations in designing / conducting the study	
Ruland	2006	Children as design partners in the development of a support system for children with cancer.	co-design	patients involved in data collection	
Santin	2019	Using a six-step co-design model to develop and test a peer-led web-based resource (PLWR) to support informal carers of cancer patients.	co-design	patients as subjects	participation
Singleton	2021	Co-designing a Lifestyle-Focused Text Message Intervention for Women After Breast Cancer Treatment: Mixed Methods Study.	experience-based co-design	patients and community representatives included in co-design	
Sun	2021	The multidisciplinary, theory-based co-design of a new digital health intervention supporting the care of oesophageal cancer patients.	co-design	patients as subjects	participation
Tang	2020	Adopting a collaborative approach in developing a prehabilitation program for patients with prostate cancer utilising experience-based co-design methodology.	experience-based co-design	patients as subjects	participation
Taylor	2014	How patients' feedback was used to redesign a head and neck service.	co-design	patients as subjects	participation
Tseng	2021	Developing a Web-Based Shared Decision-Making Tool for Fertility Preservation Among Reproductive-Age Women With Breast Cancer: An Action Research Approach.	action research	patients as subjects	participation
Tsianakas	2012	Implementing patient-centred cancer care: using experience-based co-design to improve patient experience in breast and lung cancer services.	experience-based co-design	patients as subjects	participation
vanBeusekom 2021	2021	Using Co-design With Breast Cancer Patients and Radiographers to Develop "KEW" Communication Skills Training.	co-design	patients as subjects	participation
vanBruinessen	2014	Active patient participation in the development of an online intervention.	co-design	patient partner involved in design/conducting the study	
White	2021	Engaging Carers in Co-Design: Development of the Carer Readiness Tool.	development of a tool to determine carer readiness		
Young	2020	A framework for youth-friendly genetic counseling.	framework development		
Haines	2021	Harmonizing evidence-based practice, implementation context, and implementation strategies with user-centered design: a case example in young adult cancer care.	co-design	patients on expert design committee	
Krieger	2022	Optimizing Patient Information Material for a New Psycho-Oncological Care Program Using a Participatory Health Research Approach in Germany.	participatory action research	patients on expert design committee	
